# HIV-1-associated inflammation and antiretroviral therapy regulate astrocyte endoplasmic reticulum stress responses

**DOI:** 10.1038/cddiscovery.2017.61

**Published:** 2017-12-04

**Authors:** Shruthi Nooka, Anuja Ghorpade

**Affiliations:** 1 Institute for Molecular Medicine, University of North Texas Health Science Center, Fort Worth, TX, USA

## Abstract

Antiretroviral (ARV) therapy (ART) has effectively suppressed the incidence of human immunodeficiency virus (HIV)-associated dementia in HIV-1 positive individuals. However, the prevalence of more subtle forms of neurocognitive dysfunction continues to escalate. Recently, endoplasmic reticulum (ER) stress has been linked to many neurological diseases; yet, its role in HIV/neuroAIDS remains largely unexplored. Furthermore, upregulation of astrocyte elevated gene-1 (*AEG-1*), a novel HIV-1 inducible gene, along with ER stress markers in a Huntington’s disease model, suggests a possible role in HIV-associated ER stress. The current study is focused on unfolded protein responses (UPRs) and AEG-1 regulation in primary human astrocytes exposed to HIV-associated neurocognitive disorders (HAND)-relevant stimuli (HIV-1 virions, inflammation and ARV drugs). Interleukin (IL)-1*β* and the nucleoside reverse transcriptase inhibitor abacavir upregulated expression of ER stress markers in human astrocytes, including binding immunoglobulin protein (BiP), C/EBP homologous protein (CHOP), and calnexin. In addition, IL-1*β* activated all three well-known UPR pathways: protein kinase RNA-like ER kinase (PERK); activating transcription factor 6 (ATF-6); and inositol-requiring enzyme 1*α* (IRE1*α*). AEG-1 upregulation correlated to ER stress and demonstrated astrocyte AEG-1 interaction with the calcium-binding chaperone, calnexin. IL-1*β* and abacavir enhanced intracellular calcium signaling in astrocytes in the absence of extracellular calcium, illustrating ER-associated calcium release. Alternatively, calcium evoked in response to HAND-relevant stimuli led to mitochondrial permeability transition pore (mPTP) opening in human astrocytes. Importantly, IL-1*β*- and abacavir-induced UPR and mPTP opening were inhibited by the intracellular calcium chelation, indicating the critical role of calcium signaling in HAND-relevant ER stress in astrocytes. In summary, our study highlights that ARV drugs and IL-1*β* induced UPR, AEG-1 expression, intracellular calcium, and mitochondrial depolarization in astrocytes. This study uncovers astrocyte ER stress as a novel therapeutic target in the management of HIV-1-associated neurotoxicity and possibly in the treatment of neuroAIDS.

## Introduction

Human immunodeficiency virus (HIV)-1 invades the central nervous system (CNS) during early stages of infection and often leads to neurological complications known as HIV-associated neurocognitive disorders (HAND).^1^ While the success of antiretroviral (ARV) therapy (ART) has markedly reduced the incidence of the most severe neurological manifestation, that is, HIV-associated dementia, the prevalence of cognitive dysfunctions in HIV-1-positive patients despite ART remains high.^2^ In addition to persistent low-grade viral replication and inflammation within the CNS, ARV drug toxicity also likely contributes to neurological dysfunction in HIV-1 patients.^3–5^ Consistent with this notion, withdrawal of ARV drugs in HIV-1 patients resulted in substantial improvement of neurocognitive functioning.^6,7^ Thus, ARV drugs clearly have neurotoxic effects that may contribute to HAND, necessitating the identification of underlying mechanisms and development of improved strategies for HAND treatment in the growing HIV-1 patient population.

Endoplasmic reticulum (ER) stress has been implicated in several neurological diseases, including ischemia, brain trauma, Alzheimer’s, and Parkinson’s disease.^8,9^ The ER regulates key cellular functions, including protein synthesis and folding, calcium storage, and lipid biosynthesis. Physiological and pathological stimuli, such as serum-starved conditions, high protein demand, viral infections, and inflammatory cytokines, can disrupt ER homeostasis resulting in an accumulation of misfolded proteins.^10^ To mitigate ER stress, cells activate the unfolded protein response (UPR), which is regulated by three major pathways, each with its own initiator: protein kinase RNA-like ER kinase (PERK); activating transcription factor 6 (ATF6); or inositol-requiring enzyme 1*α* (IRE1*α*). The UPR promotes cell survival by attenuating protein translation and by inducing chaperone expression, ER-associated degradation of proteins, endogenous antioxidant responses, and autophagy. However, prolonged UPR activation can lead to orchestrated cell death, that is, apoptosis.^11^

Recent studies showed amyloid beta protein accumulation in brains of HIV-1-infected individuals, indicating the potential involvement of protein misfolding and UPR activation in HIV-1 pathogenesis.^12,13^ While UPR markers, including binding immunoglobulin protein (BiP) and ATF6 were significantly elevated in the CNS of HIV-1-positive individuals,^14^ the regulation of ER stress response in HIV-1-induced neuropathogenesis is not well understood.

During HIV-1 infection, several astrocyte-associated mechanisms lead to neurotoxicity, including excitotoxicity, inflammation, and oxidative stress.^15^ These mechanisms have also been linked to ER stress in neurodegeneration.^16,17^ In addition, recent studies showed HIV-1 transactivator of transcription (Tat) and glycoprotein (gp)120 protein expression in astrocytes induced ER stress-mediated cytotoxicity, possibly contributing to HIV-associated neuropathogenesis.^18,19^ Taken together, astrocytes represent a significant therapeutic target for reestablishing CNS homeostasis.

Recently, the multifunctional oncogene, astrocyte elevated gene-1 (*AEG-1*), was shown to dysregulate glutamate clearance by excitatory amino acid transporter 2 downregulation and HIV-associated neuroinflammation via upregulation of nuclear factor-*κ*B (NF-*κ*B) pathway in astrocytes.^20,21^ AEG-1 was also elevated in a Huntington’s disease model along with UPR markers such as BiP, C/EBP homologous protein (CHOP), and regulator of ribosome synthesis 1.^22^ These studies suggest the involvement of AEG-1 in ER stress responses during neurological diseases, including HAND.

In the present study, we investigated the role of HAND-relevant stimuli, inflammation (interleukin (IL)-1*β*) and the nucleoside reverse transcriptase inhibitor (NRTI), abacavir, in the induction of ER stress in astrocytes. We demonstrated that HAND-relevant stimuli increased astrocyte cytosolic calcium, which in turn, triggered ER stress and mitochondrial depolarization. Further, we investigated specific mechanisms through which these events were interlinked thus contributing to neurodegeneration. These findings are significant as prolonged activation of UPR due to severe/chronic ER stress likely has a critical role in HIV-associated neurodegeneration.

## Results

### HAND-relevant stimuli initiate ER stress in primary human astrocytes

**Figure 1:**
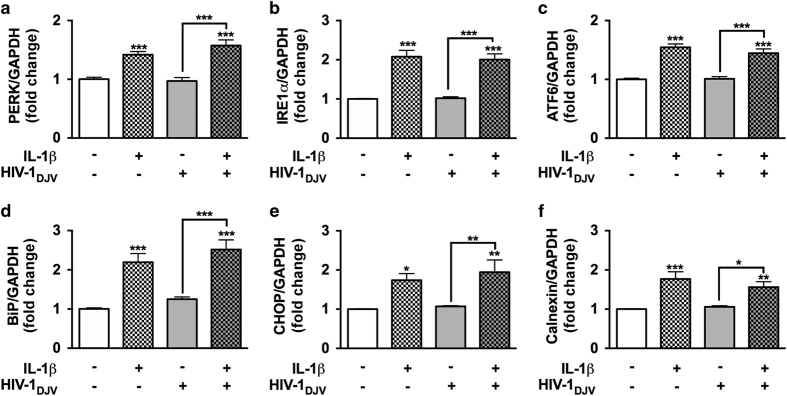
IL-1*β* with or without HIV-1 induces ER stress in astrocytes. Primary human astrocytes were treated with IL-1*β* (20 ng/ml) and HIV-1 (at 20 ng/ml p24) alone or in combination, and untreated astrocytes were maintained in parallel. After 8 h, total RNA was isolated and PERK (**a**), IRE1*α* (**b**), ATF6 (**c**), BiP (**d**), CHOP (**e**), and calnexin (**f**) mRNA levels were analyzed by RT^2^-PCR and normalized to GAPDH levels. Data represents mean fold change±S.E.M. of cumulative data from three independent donors each tested in a minimum of triplicate determinations. Statistical analyses were performed using one-way ANOVA with Tukey’s post-test for multiple comparisons (*P<0.05, **P<0.01, ***P<0.001).

To mimic a HIV-related disease environment, astrocytes were treated with IL-1*β* and HIV-1. As a common mediator of neuroinflammation, IL-1*β* is primarily released by HIV-1-infected and immune-activated microglia in the CNS.^23^ Astrocytes are highly responsive to IL-1*β* and possess an autocrine loop to perpetuate activation.^24^ Here, primary human astrocytes were incubated with IL-1*β* (20 ng/ml) and HIV-1 (p24, 20 ng/ml) alone or in combination for 8 h, and ER stress marker mRNA levels were analyzed by real-time PCR (RT^2^-PCR; Figures 1a–c). IL-1*β* alone and in combination with HIV-1 significantly upregulated three UPR proximal initiators PERK (***P<0.001; Figure 1a), IRE1*α* (***P<0.001; Figure 1b), and ATF6 mRNA levels (***P<0.001; Figure 1c). On the other hand, HIV-1 alone did not alter expression of these markers. In parallel, downstream ER stress markers BiP, CHOP, and calnexin were evaluated (Figures 1d–f). In astrocytes, IL-1*β* alone and in combination with HIV-1 significantly increased expression of BiP (***P<0.001; Figure 1d), CHOP (*P<0.05, **P<0.01; Figure 1e), and calnexin mRNA (**P<0.01, ***P<0.001; Figure 1f). However, HIV-1, by itself, did not alter mRNA levels of tested markers as compared to controls. IL-1*β* was thus identified as a key ER stress inducer in human astrocytes during HIV-associated inflammation.

### NRTIs trigger ER stress and AEG-1 expression in astrocytes

**Figure 2:**
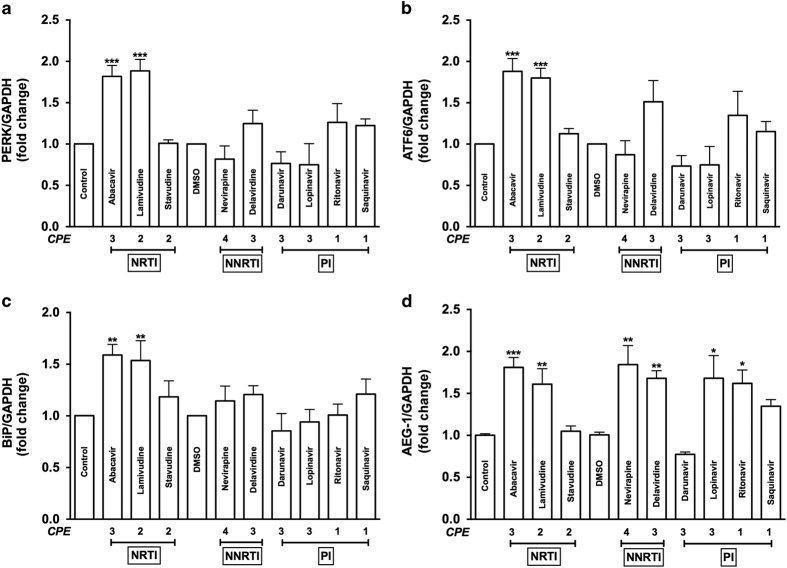
ARV drugs regulate astrocyte ER stress markers and AEG-1 mRNA levels. Astrocytes were treated with a panel of drugs, including NRTIs (abacavir, lamivudine, and stavudine), NNRTIs (nevirapine and delavirdine), and PIs (darunavir, lopinavir, and ritonavir) with CPE between 1 and 4 at therapeutic doses along with vehicle controls for 8 h. PERK (**a**), ATF6 (**b**), BiP (**c**), and AEG-1 (**d**) mRNA levels were measured using RT^2^-PCR. Fold changes to untreated control were calculated using GAPDH as a normalizing control. Cumulative data from a minimum of three independent donors are shown. Data represent mean±S.E.M., and statistical analyses were performed using one-way ANOVA with Tukey’s post-test for multiple comparisons (*P<0.05, **P<0.01, ***P<0.001).

Despite the benefits attained with ARV regimen, the ‘successful failure’ of ART is that it effectively inhibits viral replication while concurrently triggering adverse side effects and toxicities.^25,26^ However, ART-associated toxicity in the CNS, particularly in glial cells, remains largely unexplored. To investigate ARV drug-mediated ER stress, astrocytes were treated with a wide range of ARV drugs, including NRTIs (abacavir, lamivudine, and stavudine), non-nucleoside reverse transcriptase inhibitors (NNRTIs; nevirapine and delavirdine), and protease inhibitors (PIs; darunavir, lopinavir, ritonavir, and saquinavir) at therapeutic doses (Figure 2). These ARV drugs were used clinically and have CNS penetration effectiveness (CPE) scores ranging from 1 to 4, with higher scores reflecting greater likelihood of CNS penetration.^27^ ER stress marker mRNA levels were evaluated 8 h post-treatment by RT^2^-PCR. Levels of PERK (***P<0.001; Figure 2a), ATF6 (***P<0.001; Figure 2b), and BiP mRNA (**P<0.01; Figure 2c) were significantly increased with abacavir or lamivudine treatments. The NNRTI delavirdine increased mRNA levels by ~1.5-fold, which was not statistically significant for PERK, ATF6, and BiP (Figures 2a–c). NRTIs (abacavir and lamivudine), NNRTIs (nevirapine and delavirdine), and PIs (lopinavir and ritonavir) significantly elevated AEG-1 mRNA as compared to respective controls (*P<0.05, **P<0.01, and ***P<0.001; Figure 2d). However, stavudine, darunavir, and saquinavir did not increase AEG-1 expression. As AEG-1 mRNA levels were elevated by ARV drugs with greater CNS penetration (i.e., abacavir, CPE 3; nevirapine, CPE 4; delavirdine, CPE 3; and lopinavir, CPE 3), exploring the effects of ART on AEG-1-regulated neurological outcomes is therapeutically relevant. Taken together, of all the ARV drugs tested, only abacavir and lamivudine triggered both UPR markers and AEG-1 upregulation. Therefore, considering its high CPE and current clinical use, abacavir was used to further study how ARV drugs regulate astrocyte ER stress.

### HAND-relevant stimuli activate UPR and promote selective protein translation

**Figure 3:**
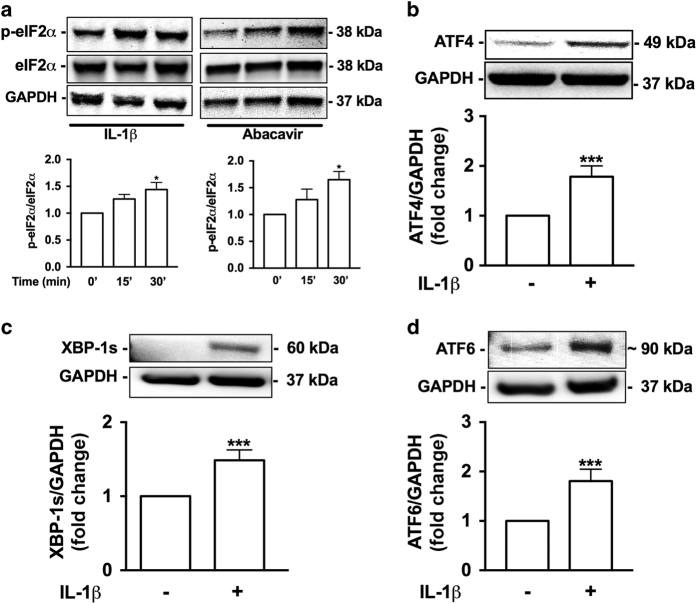
IL-1*β* and abacavir activate UPR and trigger eIF2*α* phosphorylation. Astrocytes were treated with IL-1*β* (20 ng/ml) or abacavir (4 *μ*M) for 15 and 30 min. Total protein lysates were immunoblotted for p-eIF2*α* and eIF2*α* (**a**). Densitometry analyses were performed to quantify the intensity ratio of p-eIF2*α* to total eIF2*α*, which are graphed as fold change to control. One-way ANOVA with Tukey’s post-test was used to determine statistical significance (*P<0.05). Whole-cell lysates of astrocytes treated with IL-1*β* (20 ng/ml) for 6 h were immunoblotted for ATF4 (**b**), XBP-1s (**c**), and ATF6 (**d**). Statistical analyses were performed by Student’s unpaired *T*-test to compare fold change to control (***P<0.001). In all panels, representative blots are presented, while the average fold change from three independent donors is graphed. GAPDH was used as a normalizing loading control.

The three distinct UPR pathways, PERK, IRE1*α*, and ATF6, can activate transcription of ER stress response element (ERSE)-regulated genes, including *BiP* and *CHOP*. Therefore, we next focused on identifying which specific UPR mechanism(s) regulate ER stress in astrocytes during inflammation and abacavir exposure. Western blot analysis confirmed increased eIF2*α* phosphorylation following IL-1*β* or abacavir treatment (*P<0.05; Figure 3a), suggesting that general attenuation of protein translation in astrocytes occurred via PERK activation. PERK-mediated eIF2*α* phosphorylation supports translation of ATF4, an ERSE transcription factor, normally suppressed by an inhibitory open reading frame. When active, eIF2*α* is abundant, and selective translation of ER stress mitigating genes is promoted.^28^ Our studies confirmed IL-1*β* significantly increased ATF4 levels in astrocytes (***P<0.001; Figure 3b). Next, to evaluate IRE1*α*- and ATF6-mediated UPR activation, whole-cell lysates were immunoblotted for the spliced form of XBP-1 (XBP-1s) and cleaved ATF6 (Figures 3c and d), the respective UPR-associated ERSE transcription factors. IL-1*β* significantly increased XBP-1s (***P<0.001; Figure 3c) and ATF6 (***P<0.001; Figure 3d) as compared to controls. Together, these results indicate that HAND-relevant stimuli induce transcriptional regulation during ER stress in astrocytes via each of the three UPR pathways: PERK, IRE1*α*, and ATF6.

### IL-1*β* and abacavir transiently induce intracellular calcium in GCaMP6-transfected astrocytes

**Figure 4:**
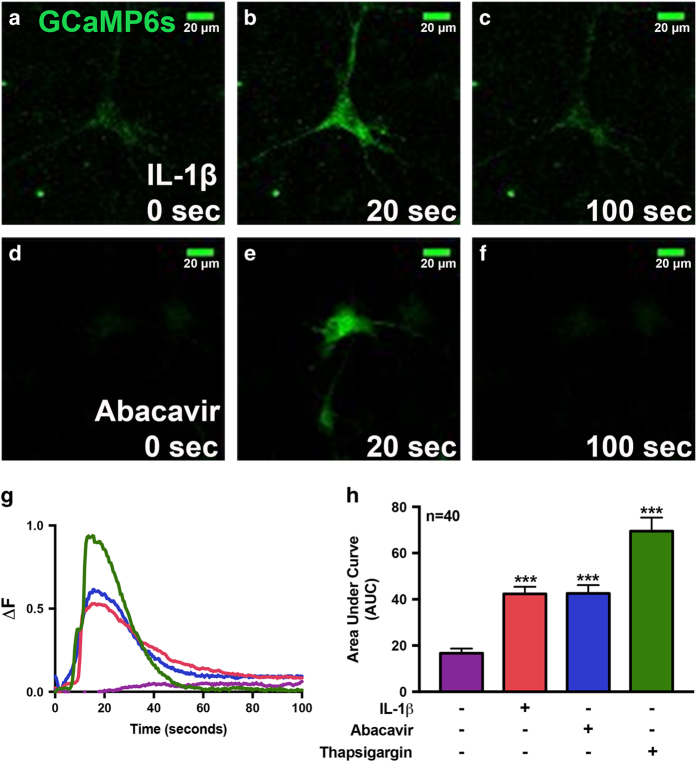
IL-1*β* and abacavir induce intracellular calcium signaling in human astrocytes. For analysis of calcium signaling, primary human astrocytes were first transfected with GCaMP6s, a plasmid expressing an ultrasensitive protein calcium sensor, and allowed to recover for 48 h. Transfected cells were treated with IL-1*β* (20 ng/ml, **a**–**c**) and abacavir (4 *μ*M, **d**–**f**). Fluorescence was visualized by confocal microscopy and images were captured every 500 ms. (**a**–**f**) Fluorescent images taken from a representative cell at time 0 (**a** and **d**, before treatment) and at 20 (**b** and **e**) and 100 s (**c** and **f**), post IL-1*β* and abacavir treatment, respectively. The histogram (**g**) shows fluorescence intensity ratio (Δ*F*) of the representative astrocyte captured over the entire imaging period before and after treatments with HBSS (control, violet), IL-1*β* (orange), abacavir (blue), and Th (200 nM, green). Bar graph (**h**) denotes intracellular Ca^+2^ increase, quantified as area under the curve (AUC) in IL-1*β*-, abacavir-, and Th-treated astrocytes as compared to control. Cumulative data from three individual donors are shown. Data represent mean±S.E.M., and statistical analyses were performed using one-way ANOVA with Tukey’s post-test for multiple comparisons (***P<0.001, *n*=40 individual cells/treatment).

To investigate IL-1*β*- and abacavir-induced intracellular calcium ([Ca^+2^]_i_) signaling, primary human astrocytes were first transfected with GCaMP6 slow (GCamp6s) plasmid, a genetically encoded calcium sensor,^29^ with an ~80% transfection efficiency. Intracellular calcium was visualized by live, fluorescent, confocal imaging of GCaMP6s-transfected astrocytes. Baseline fluorescence was observed at 0 s (Figures 4a and d), which increased at 20 s after IL-1*β* and abacavir treatment (Figures 4b and e). Over time, fluorescence decreased returning to baseline by ~100 s (Figures 4c and f). Changes in fluorescence were reflective of calcium transients (Δ*F*=(*F*−*F*_0_)/(*F*_max_−*F*_0_), where *F* is the fluorescence intensity at any given time, *F*_max_ is the maximum fluorescence intensity, and *F*_0_ is the baseline fluorescence intensity). Calcium changes induced with IL-1*β*, abacavir, and thapsigargin (Th), a known ER stressor, were plotted from representative astrocytes over time (Figure 4g). The dynamic changes in calcium transients were quantified using area under the curve to assess total change in calcium levels in a given astrocyte and to compare responses across treatments. IL-1*β*, abacavir, and Th each elicited significantly higher average [Ca^+2^]_i_ than control (***P<0.001; Figure 4h, *n*=40 individual cells/treatment). Thus, these data validate that IL-1*β* and abacavir triggered transient intracellular calcium increase in astrocytes, comparable to that induced by Th.

### HAND-relevant stimuli increase ER quality control mechanism in astrocytes

**Figure 5:**
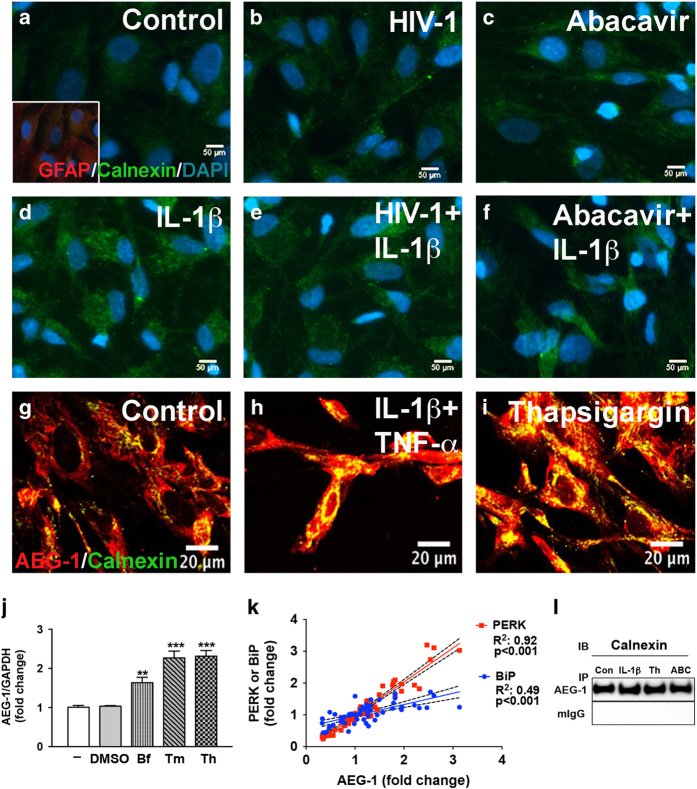
HAND-relevant stimuli increase calnexin levels and colocalization with AEG-1 in the context of ER stress. Astrocytes were treated with IL-1*β* (20 ng/ml) alone, HIV-1 (p24, 20 ng/ml)±IL-1*β*, and abacavir (4 *μ*M)±IL-1*β* for 24 h. Representative calnexin (green) and GFAP (red) immunofluorescent images of untreated (**a**), HIV-1 (**b**)-, abacavir (**c**)-, IL-1*β* (**d**)-, HIV-1+IL-1*β* (**e**)-, and abacavir+IL-1*β* (**f**)-treated astrocytes were shown. Original magnification ×200. AEG-1 and calnexin (yellow) perinuclear colocalization was confirmed by confocal analysis of astrocytes (**g**) treated with IL-1*β*+TNF-*α* (**h**), and Th (**i**) for 24 h. Cultures were fixed and immunostained for AEG-1 (red) and calnexin (green), which are shown as summed *Z*-stacked images of 12 micrographs taken at 0.5 *μ*m intervals. Original magnification ×400. AEG-1 mRNA expression was assessed by RT^2^-PCR (**j**) following 8 h treatment with vehicle or the known ER stressors, Bf (1 *μ*g/ml), Tm (1 *μ*g/ml), and Th (200 nM). Cumulative data represent mean±S.E.M., and statistical analyses were performed using one-way ANOVA with Tukey’s post-test for multiple comparisons (**P<0.01, ***P<0.001). Experiment was conducted in three individual donors. AEG-1 expression positively correlated to PERK (*R*^2^=0.92, ***P<0.001) and BiP (*R*^2^=0.49, ***P<0.001) (**k**) in the ARV drug-treated astrocytes discussed in Figure 2. To evaluate AEG-1 and calnexin interaction, lysates from 24 h untreated, IL-1*β*-, Th-, and abacavir (ABC)-treated astrocytes were coimmunoprecipitated with AEG-1 antibody or mouse IgG, and immunoblotted for calnexin (**l**). Representative data are shown from three astrocyte donors that were tested.

During ER stress, UPR activation increases chaperones such as BiP, calnexin, and calreticulin to promote proper protein folding and quality control in the ER.^30^ HAND-relevant stimuli increased calnexin levels in astrocytes (Figure 5). Astrocytes were treated with HIV-1 (Figure 5b), abacavir (Figure 5c), IL-1*β* (Figure 5d), HIV-1+IL-1*β* (Figure 5e), and abacavir+IL-1*β* (Figure 5f) for 24 h. Cells were fixed and immunostained for glial fibrillary acidic protein (GFAP; red), calnexin (green), and DAPI (blue). Untreated astrocytes retained bright GFAP with lower perinuclear calnexin staining (Figure 5a). HAND-relevant stimuli (Figures 5b–f) increased calnexin expression in astrocytes.

### AEG-1 colocalizes and interacts with calnexin in the context of ER stress

As a pleotropic protein, AEG-1 can localize to the cell membrane, cytoplasm, ER, nucleus, and nucleolus, and exhibits diverse location-related functions.^31^ AEG-1 colocalized with calnexin in the ER, which was increased with IL-1*β*+tumor necrosis factor (TNF)-*α*, and Th treatment (Figures 5g–i) when assessed by confocal microscopy. To study how ER stress influences AEG-1, astrocytes were treated with ER stress inducing agents brefeldin A (Bf), tunicamycin (Tm), and Th followed by RT^2^-PCR analysis (Figure 5j). These ER stressors significantly upregulated AEG-1 mRNA levels as compared to controls (**P<0.01, ***P<0.001; Figure 5j). Both Tm and Th upregulated AEG-1 by 2.5-fold (Figure 5j), a feature that is rather uncommon for AEG-1, which primarily exhibits its function via subcellular localization and redistribution.^31^ Moreover, AEG-1 transcripts correlated significantly to BiP (*R*^2^: 0.49, ***P<0.001; Figure 5k) and PERK (*R*^2^: 0.92; ***P<0.001; Figure 5k) in the ARV drug-exposed astrocytes (shown earlier in Figure 2). AEG-1 is also known to exhibit diverse functions by interacting with several cellular proteins in cancer.^32^ As AEG-1 can function as a transcriptional coactivator and as a scaffolding protein, we examined whether AEG-1 directly binds to calnexin (Figure 5l). Consistent with the confocal analyses, coimmunoprecipitation data confirmed AEG-1 interacted with calnexin in untreated, IL-1*β*-, abacavir-, or Th-treated astrocytes (Figure 5l). These results establish that AEG-1 expression is elevated during HAND-associated ER stress. Furthermore, a direct interaction with the calcium-binding chaperone calnexin indicates that AEG-1 likely regulates ER quality control and possibly calcium signaling in astrocytes.

### HAND-relevant stimuli lead to mitochondrial permeability transition pore opening in astrocytes

**Figure 6:**
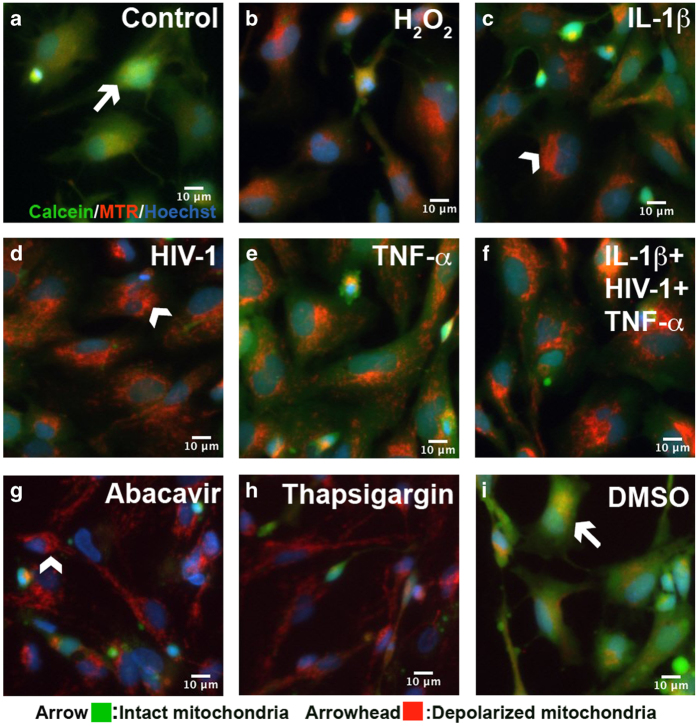
HIV-1, inflammation, oxidative stress, and ARV drugs induce astrocyte mitochondrial depolarization. Astrocytes untreated (**a**) and DMSO (vehicle; (**i**)) were treated for 24 h with H_2_O_2_ (200 *μ*M) (**b**), IL-1*β* (20 ng/ml) (**c**), HIV-1 (p24 20 ng/ml) (**d**), TNF-*α* (50 ng/ml) (**e**), IL-1*β*+HIV-1+TNF-*α* (**f**), abacavir (4 *μ*M) (**g**), and Th (200 nM) (**h**). Live-cell imaging was performed using calcein/cobalt chloride quenching assay to monitor mPTP opening. Calcein (green) colocalization with MTR (red) represents closed mPTP (arrow; green, yellow), while loss of green fluorescence represents mPTP opening (arrowhead, red). Representative images from three donors are shown. Original magnification ×200 Th, Thapsigargin.

Cross talk between neurodegenerative mechanisms, including inflammation, oxidative, and ER stress, increases the severity of many diseases.^17^ A persistent increase in [Ca^2+^]_i_ in response to pathophysiological stimuli can lead to mitochondrial permeability transition pore (mPTP) opening, which compromises mitochondrial function by eliminating membrane electrochemical potential and increasing reactive oxygen species.^33^ Therefore, mPTP opening in response to HAND-relevant stimuli, oxidative stress, and ARV drugs was monitored using a fluorescent live mitochondrial transition pore assay (Figure 6). Astrocytes were stained with the nuclear indicator Hoechst (blue), the mitochondrial dye mitotracker red (MTR) and calcein-AM, a membrane permeable fluorophore that diffuses freely into mitochondria and is quenched by cobalt chloride in the cytoplasm. Using live-cell fluorescent imaging, calcein and MTR labeling were measured in astrocytes (Figure 6a) treated with H_2_O_2_ (200 *μ*M, Figure 6b), IL-1*β* (20 ng/ml, Figure 6c), HIV-1 (p24, 20 ng/ml, Figure 6d), TNF-*α* (50 ng/ml, Figure 6e), IL-1*β*+HIV-1+TNF-*α* together (Figure 6f), abacavir (4 *μ*M, Figure 6g), Th (100 nM, Figure 6h), and DMSO (vehicle for Th, Figure 6i). Untreated and vehicle-treated astrocytes exhibited calcein (green) and MTR (red) colocalization in mitochondria (yellow; Figures 6a and i, arrows), reflecting closed mPTP. Calcein fluorescence was decreased in astrocytes treated with H_2_O_2_ and other HAND-relevant stimuli (Figures 6b–h), indicating mitochondrial pore opening (Figure 6, arrowhead). Together, these data show that HAND-relevant stimuli deleteriously affected astrocytes by promoting mPTP opening and loss of mitochondrial membrane integrity.

### Intracellular calcium chelation inhibits abacavir- and IL-1*β*-mediated ER stress and subsequent mitochondrial damage in human astrocytes

**Figure 7:**
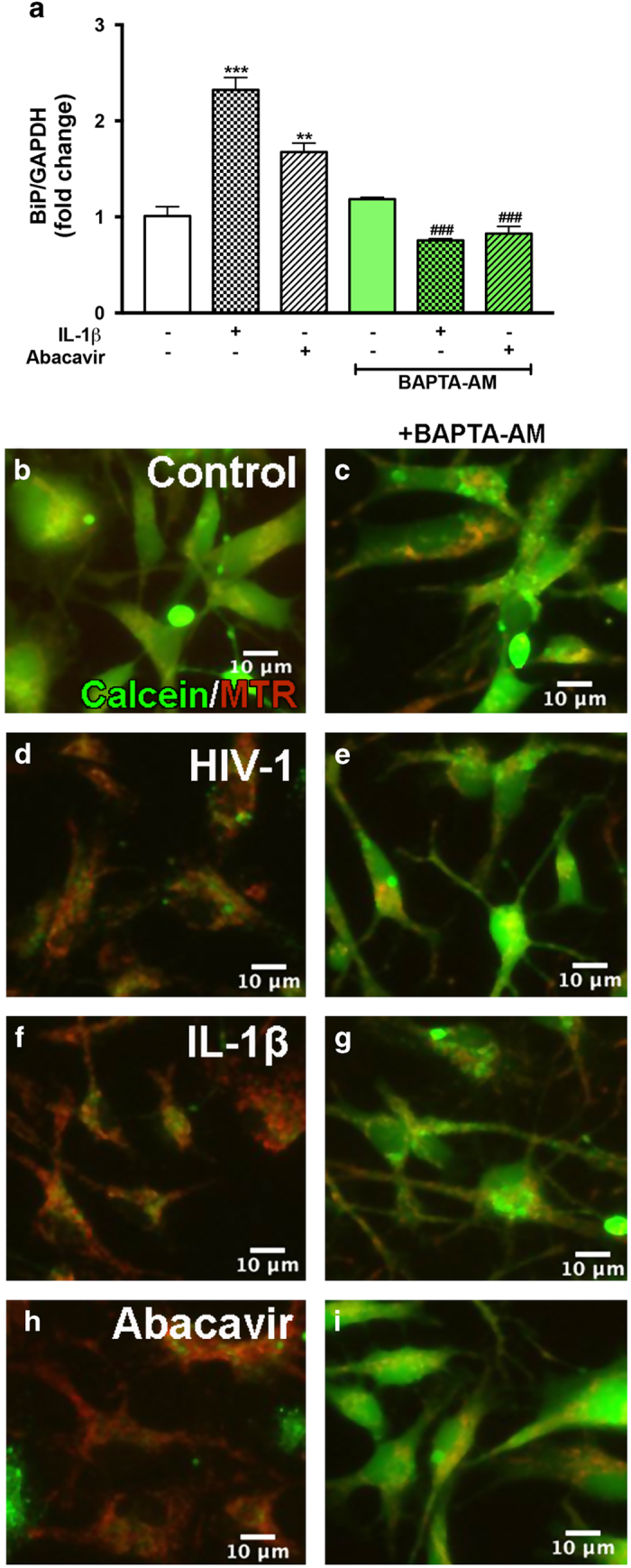
Chelating intracellular calcium induced by IL-1*β* and abacavir reduces ER stress and mitochondrial depolarization. Astrocytes were treated with IL-1*β* (20 ng/ml), HIV-1 (p24 20 ng/ml), and abacavir (4 *μ*M) for 8 or 24 h with or without pre-treatment with BAPTA-AM (25 *μ*M) for 1 h. Total RNA was extracted after 8 h and BiP mRNA expression, as a measure of ER stress, was determined by RT^2^-PCR (**a**). Cumulative data from three individual donors are shown. Data represent mean±S.E.M. Statistical significance was determined by one-way ANOVA with Tukey’s post-test for multiple comparisons (**P<0.01, ***P<0.001, ^###^*P*<0.001), where # represents statistical significance for treatment±BAPTA-AM comparisons. (**b**–**i**) Live-cell fluorescent images for mPTP detection by calcein/cobalt chloride assay. Representative calcein (green) and MTR (red) fluorescence images from one of three donors are shown from untreated (**b**), BAPTA-AM pre-treated (**c**), HIV-1 (**d**)-, HIV-1+BAPTA-AM (**e**)-, IL-1*β* (**f**)-, IL-1*β*+BAPTA-AM (**g**), abacavir (**h**)-, and abacavir+BAPTA-AM (**i**)-treated astrocytes. Original magnification ×200.

To examine the effects of IL-1*β*- and abacavir-induced calcium release on ER stress and mPTP opening, astrocytes were pre-treated with the calcium chelator, 1,2-bis (2-aminophenoxy) ethane-*N*,*N*,*N*',*N*'-tetraaceticacid (acetoxymethyl ester) (BAPTA-AM). Consistent with Figures 1 and 2, BiP was significantly increased with IL-1*β* and abacavir alone (**P<0.01, ***P<0.001; Figure 7a). In parallel, BAPTA-AM pre-treatment successfully decreased IL-1*β*- and abacavir-induced BiP expression (^###^*P*<0.001; Figure 7a). To identify the consequences of elevated [Ca^2+^]_i_ on mitochondrial membrane integrity, live-cell fluorescent imaging for calcein-AM (green) and MTR (red) was performed. Loss of calcein (green) and increase in MTR (red) fluorescence were observed in HIV-1-, IL-1*β*-, and abacavir-treated astrocytes (Figures 7d, f and h). However, BAPTA-AM pre-treatment preserved mitochondrial integrity as indicated by calcein fluorescence (green) in control and HAND-relevant stimuli-treated astrocytes (Figures 7b, c, e, g and i). These results demonstrate that IL-1*β*- and abacavir-induced ER stress and mPTP opening are highly calcium-dependent.

## Discussion

Neurological impairments continue to be a major health concern for HIV-infected patients in the post-ART era.^1^ Nevertheless, indirect causes and targetable mechanisms of neuronal degeneration in HAND are not well understood. ER stress is prevalent in several neurodegenerative diseases, though its regulation and contributions to neuropathogenesis have not been well studied in HAND.^8,9^ Aiming for a better understanding of HIV-1-induced UPR activation, the present study is focused on CNS neuroglial cells, that is, astrocytes, with various HAND-relevant stimuli (HIV-1, ARV drugs, and inflammation). Our results demonstrate that HAND-relevant stimuli induce ER stress and activate all three UPR pathways: PERK, IRE1*α*, and ATF6 in human astrocytes. Further, astrocyte intracellular calcium is elevated by IL-1*β* and abacavir via ER calcium release, which, when prolonged, triggers ER stress responses and mitochondrial depolarization. We also document elevated AEG-1 expression that positively correlates with ART-mediated ER stress. AEG-1 colocalization and interaction with calnexin implicate AEG-1 as a scaffolding protein regulating calcium signaling and ER function.

Ongoing low-level CNS viral replication and HIV-1-induced inflammation contribute to HAND.^3–5^ Inflammatory cytokines have an important role in pathogenesis of HIV-1-associated neurocognitive impairments. Several cytokines, including IL-1*β* and TNF-*α* are dysregulated in encephalitic brains of HIV-1-infected patients.^23,34,35^ Many of these elements can initiate ER stress response in astrocytes.^10^ However, the molecular mechanisms that underlie HAND-relevant stimuli-mediated ER stress in astrocytes remain unknown.

Our findings demonstrate that HAND-associated inflammation induce ER stress in astrocytes. ER stress may be both a trigger and consequence of inflammation in many chronic inflammatory diseases.^36^ According to recent studies, ER stress-induced UPR signaling is associated with the production of many proinflammatory molecules.^37^ All three UPR pathways transcriptionally regulate the expression of inflammatory molecules through NF-*κ*B and activator protein.^36,38,39^ Our study identifies IL-1*β* as a strong initiator of ER stress and the UPR in astrocytes, highlighting cross talk between ER stress and inflammation during HIV-1 CNS infection.

Recently, the CNS penetrance of ARV drugs has become increasingly clinically significant as the battle to reduce HIV-1-mediated neurotoxicity.^40^ However, long-term ART is associated with a range of adverse effects, including neuropathy, hyperbilirubinemia, lipodystrophy, neuropsychiatric disorders, retinal lesions, and hypersensitivity.^41^ ARV drugs triggered adverse side effects and toxicities, including, but not limited to, alterations in lipid and protein metabolism, insulin resistance, mitochondrial toxicity, oxidative stress, and neuronal damage.^25,26^ An *in vitro* study evaluated the neurotoxic effects of several ARV drugs on rat forebrain neuronal cultures. Out of the various ARV classes tested, NRTIs, including abacavir, showed high neurotoxicity.^42^ Therefore, another possible contributor to HAND is ARV drug-related neurotoxicity.

Our results demonstrate increased mRNA levels of ER chaperones, BiP and CHOP, with HAND-relevant stimuli, indicating ER stress in primary human astrocytes. While the UPR elevates BiP and CHOP expression, each has an opposing role in the outcome of the ER stress response. As a pro-survival protein, BiP removes ER misfolded proteins; whereas CHOP promotes apoptosis by favoring mitochondrial depolarization, cytochrome c release, and caspase 3 activation.^43^ Thus, the cell fate depends on the critical balance between BiP and CHOP.^44^ Initially, HAND-relevant stimuli significantly upregulated both BiP and CHOP. At later time points, BiP expression was decreased (data not shown), indicating that the disrupted balance between these two proteins may lead to cell death or apoptosis. Furthermore, cortical autopsy tissues from HIV-1-infected patients showed upregulated ER stress markers BiP and ATF6 in various CNS cell types, including neurons and astrocytes.^14,45^ Therefore, further investigation of ER stress-associated neurotoxic mechanisms of ARV drugs alone or in combination is warranted.

ER stress can induce three independent UPR signaling pathways: PERK, IRE1*α*, and ATF6. Elevation of all proximal sensors and downstream transcriptional factors suggests that all three UPR pathways regulate HAND-associated ER stress in astrocytes. In addition, IL-1*β* and abacavir increased eIF2*α* phosphorylation, indicating general attenuation of protein translation; a strategy to decrease the load of newly synthesized proteins in the ER lumen. However, UPR activation of ERSE-binding transcription factors, including ATF4, XBP-1s, and ATF6, upregulates compensatory expression of ER stress regulatory proteins, including BiP and CHOP.

One study evaluated AEG expression, including AEG-1 and AEG-9 (a.k.a. calnexin), following HIV-1 infection or treatment with gp120 in astrocytes.^46^ Recently, we identified AEG-1 as a novel modulator of HIV-1-associated neuroinflammation and glutamate clearance in astrocytes, suggesting a significant role in HAND.^20,21^ Therefore, we investigated plausible AEG-1 contributions toward HIV-1/ART-induced ER stress in astrocytes. The present study identifies *AEG-1* as an ER stress inducible gene in astrocytes. To our knowledge, this is the first report to recognize AEG-1 as an interacting partner of calnexin, and reinforces AEG-1 as a scaffolding protein regulating ER calcium signaling through formation of multi-protein complexes.

ER harbors an intracellular calcium pool, which regulates essential cellular functions via calcium signaling. The present study demonstrates that HAND-relevant stimuli increase intracellular calcium in astrocytes. In addition, other studies have shown that HIV-1 proteins disrupt neuronal calcium homeostasis during HIV-1 encephalitis.^47,48^ Many ER calcium-binding proteins, including calreticulin, calnexin, BiP, and GRP94 have large calcium-binding capacities and regulate ER calcium homeostasis. Increased calnexin expression supports the theory that HAND-relevant stimuli fluctuate ER calcium in astrocytes. Severe disruption of ER calcium homeostasis also triggers ER stress and UPR signaling pathways to upregulate ER stress markers.^49^ Our study also shows upregulated expression of calcium-binding chaperones such as BiP. However, in addition to its chaperone function, BiP also has an important role in the intraluminal storage of calcium.^50^

The ER serves as the principal calcium store in the cells, and dynamic calcium transfer through ER–mitochondria contact sites leads to various cellular coordinated responses. Calcium-induced mPTP opening and permeabilization of mitochondrial membrane are the key events in early apoptosis.^51^ Prolonged mPTP opening has a crucial role in the pathogenesis of several diseases.^52^ Intracellular calcium chelation significantly reduced mPTP opening, suggesting that HAND-relevant stimuli-induced calcium influx into mitochondrial matrix causing mitochondrial dysfunction. Further, increased intracellular calcium can also induce calcium-dependent exocytosis, leading to excessive glutamate release from astrocytes.^53^ Other studies also support this conclusion as buffering of intracellular calcium reduced glutamate release in astrocytes.^54^ Excessive synaptic glutamate causes excitatory neurotransmission resulting in neuronal damage.

**Figure 8:**
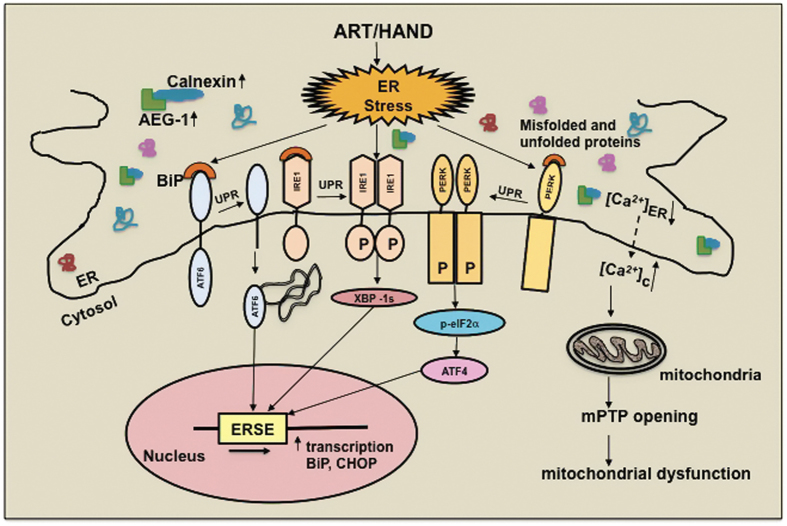
HIV-associated inflammation and therapy induce ER stress and UPR signaling in astrocytes. HAND-relevant inflammatory stimuli induce ER stress and trigger UPR activation in astrocytes. Increased ATF4, XBP-1s, and ATF6 levels result in the transcriptional activation of ERSE-regulated genes such as *BiP* and *CHOP*. IL-1*β* and abacavir increase expression of calnexin, a calcium-binding ER chaperone. Concurrently, AEG-1 expression is augmented, this coupled with its interactions with calnexin possibly acting as a scaffolding protein, implicating it in regulation of ER stress and calcium regulation. Here we show that IL-1*β*- and abacavir-mediated increases in intracellular calcium trigger ER stress and mitochondrial dysfunction. As calcium chelation blocks IL-1*β*- and abacavir-mediated changes in BiP and mPTP opening, calcium appears to be a critical regulator of HAND-relevant ER stress responses. Taken together, perturbations in astrocyte calcium regulation, ER and mitochondrial function by ART and HAND-relevant inflammation may have significant mechanistic roles in HIV-1-associated neuropathogenesis.

Taken together, HIV-1-associated inflammation and ART lead to ER stress and activation of UPR signaling in astrocytes. Our findings in the present study for the first time demonstrate that IL-1*β* and the NRTI abacavir induce intracellular calcium dysregulation, ER stress, AEG-1 expression, and mitochondrial dysfunction (mPTP opening) in astrocytes (Figure 8). In addition to deciphering these outcomes as independent correlates of HAND-relevant astrocyte activation, our studies also delineated the temporal order of these events and their concerted roles in astrocyte ER stress as summarized in Figure 8. We propose that in the post-ART era, chronic UPR activation and associated ER stress in astrocytes likely have key roles in mediating neurotoxicity in HIV-1-infected patients. In particular, these findings uncover astrocyte ER stress as a unique opportunity to explore novel therapeutic targets during HIV-1-associated neuropathogenesis.

## Materials and methods

### Isolation, cultivation, and treatment of primary human astrocytes

Human astrocytes were isolated from first- and early second-trimester-aborted specimens as previously described.^55^ Tissues were obtained in full compliance with ethical guidelines of the University of Washington, the University of North Texas Health Science Center, and the National Institutes of Health. Briefly, brain tissues were dissected and mechanically dissociated. Cell suspensions were centrifuged, washed, suspended in media, and plated at a density of 20-50×10^6^ cells/150 cm^2^. Adherent astrocytes underwent several passages to enhance the purity of replicating astroglial cells before experimental use. The astrocyte preparations were routinely >99% pure as measured by immunocytochemical staining for GFAP and microglial marker CD68 to determine possible microglial contamination and contribution of microglia in inflammatory responses. Astrocytes were treated with IL-1*β* (20 ng/ml, R&D Systems, Minneapolis, MN, USA); HIV-1_DJV_ (p24, 20 ng/ml); TNF-*α* (50 ng/ml, R&D Systems); ER stress compounds, Th (0.2 nM, Cell Signaling, Baltimore, MD, USA), Tm (1 *μ*g/ml, Cell Signaling), and Bf (0.5 *μ*g/ml, Cell Signaling); ARV drugs: abacavir (4 *μ*M, ChemPacific, Baltimore, MD, USA), saquinavir (22.5 *μ*M, ChemPacific), delavirdine (22.5 *μ*M, ChemPacific), lopinavir (0.015 *μ*M, ChemPacific), darunavir (5 nM, ChemPacific), lamivudine (11 *μ*M, ChemPacific), stavudine (2.25 *μ*M, ChemPacific), nevirapine (0.1 *μ*M, ChemPacific), and ritonavir (0.1 *μ*M, ChemPacific); and the calcium chelator BAPTA-AM (25 *μ*M, Sigma-Aldrich, St Louis, MO, USA) at 37 °C and 5% CO_2_. HIV-1_DJV_ was originally isolated from monocyte cultivation from a HIV-1-demented human brain tissue and was subsequently expanded in culture in peripheral blood monocytes.^56^

### RNA extraction and gene expression analyses

Astrocyte RNA was isolated 8 h post treatment as described previously.^57^ RNA was reverse transcribed into cDNA as per the manufacturer’s instructions, and gene expression was assayed by RT^2^-PCR. Taqman 5′ nuclease RT^2^-PCR was performed using StepOnePlus detection system (Thermo Fisher Scientific, Waltham, MA, USA). Commercially available TaqMan gene expression assays were used to measure PERK (catalog no. HS00984006_m1), IRE1*α* (catalog no. Hs00176385_m1), ATF6 (catalog no. Hs00232586_m1), BiP (Hs00607129_gH), CHOP (catalog no. Hs00358796), calnexin (catalog no. Hs01558409_m1), AEG-1 (catalog no. Hs00757841_m1), and GAPDH (catalog no. 4310884E, Thermo Fisher Scientific) mRNA. *GAPDH*, a ubiquitously expressed housekeeping gene, was used as an internal normalizing control. The 25 *μ*l reactions were carried out at 48 °C for 30 min, 95 °C for 10 min, followed by 40 cycles of 95 °C for 15 s and 60 °C for 1 min in 96-well optical, RT^2^-PCR plates. Transcripts were quantified by the comparative ΔΔCT method and represented as fold change to control.

### Western blot analyses

Astrocytes were cultured as adherent monolayers in 75 cm^2^ flasks at a density of 8×10^6^ cells/flask and allowed to recover for 24 h. Following recovery, cells were treated for various time points, and whole-cell protein extracts were isolated using mammalian protein extraction buffer (Thermo Fisher Scientific). Cells were collected by scraping in sterile ice-cold PBS to avoid alteration of protein expression on surface of cell membranes. Whole-cell protein extracts (25 *μ*g) were boiled with 4× NuPAGE loading sample buffer at 100 °C for 5 min, resolved by Bolt 4–12% bis tris gel and subsequently transferred to nitrocellulose membranes using i-Blot (Thermo Fisher Scientific). The membranes were incubated with antibodies against ATF4 (1 : 1000, Cell Signaling), ATF6 (1 : 1000, Cell Signaling), XBP-1s (1 : 1000, Cell Signaling), p-eIF2*α*, and eIF2*α* (1 : 1000, Cell Signaling) overnight at 4 °C, washed, and then incubated with anti-rabbit IgG conjugated to horseradish peroxidase (1 : 10 000, Bio-Rad, Hercules, CA, USA) or anti-mouse IgG conjugated to horseradish peroxidase (1 : 10 000, Bio-Rad) for 2 h at room temperature. The membranes were then developed with SuperSignal west femto substrate (Thermo Fisher Scientific) and imaged using Fluorochem HD2 imager (ProteinSimple, San Jose, CA, USA). GAPDH (1 : 2000, Santa Cruz Biotechnology, Dallas, TX, USA) was used as a loading control.

### Immunocytochemistry

Astrocytes were cultured as adherent monolayers at a density of 0.1×10^6^ cells/well. Cultured cells were fixed after 24 h treatment with ice-cold acetone:methanol (1 : 1) solution for 20 min at −20 °C and blocked with blocking buffer (2% BSA in 1× PBS containing 0.1% Triton X-100) for 1 h at room temperature. Cells were then incubated with primary antibodies specific to calnexin (1 : 500, Cell Signaling), GFAP (1 : 400, Covance Inc., Princeton, NJ, USA) in blocking buffer overnight at 4 °C, washed, and incubated with AlexaFluor secondary antibodies, anti-rabbit (488 nm), and anti-chicken (594 nm; 1 : 100, Thermo Fisher Scientific). Micrographs were obtained on an ECLIPSE Ti-4 using the NIS-Elements BR. 3.2 software (Nikon, Minato, Tokyo, Japan).

### Transfection of astrocytes

Cultured human astrocytes were transfected with pGP-CMV-GCaMP6s, a gift from Douglas Kim (Addgene, Cambridge, MA, USA; plasmid # 40753)^29^ using the Amaxa P3 primary cell 96-well kit, nucleofector and shuttle attachment (Lonza, Walkersville, MD, USA) according to the modified, manufacturer’s instructions. Briefly, 1.6×10^6^ astrocytes were suspended in 20 *μ*l nucleofector solution containing GCaMP6s (0.5 *μ*g) and were electroporated using shuttle protocol CL133. Transfected cells were supplemented with astrocyte media and incubated for 30 min at 37 °C before plating into six channel *μ*-slides (0.4 VI, ibidi, Madison, WI, USA) at 1×10^5^/channel. Cells were allowed to recover for 48 h before confocal imaging. Minimums of three channels were imaged from each biological astrocyte donor per treatment condition.

### Confocal analyses

For colocalization studies, human non-transfected or transfected astrocytes were cultured on glass-bottom 48-well tissue culture plates (MatTek Corp., Ashland, MA, USA) at 1×10^5^ cells/well in astrocyte media for 48 h. Cells were then treated for 24 h and carefully fixed before staining with antibodies specific to AEG-1 (1 : 200, Thermo Fisher Scientific) and calnexin as described in ICC. Before live-cell calcium imaging, astrocytes in *μ*-slides were briefly washed with PBS and supplemented with phenol red-, calcium-, and magnesium-free Hank’s buffered saline solution at 37 °C. Time-lapse micrographs were acquired every 500 ms for 6 min from astrocytes treated with IL-1*β* (20 ng/ml), abacavir (4 *μ*M), and ionomycin (10 *μ*M) at 10 s. Micrographs were obtained on Carl Zeiss LSM (Jena, Germany). The objective used was ×20 Plan-Apochromat, 0.8 NA, 0.55 mm WD. Photomultiplier tube detection was used with an excitation of 450–490 nm and emission of 593–668 nm. Colocalization, live-cell video imaging, and histogram analyses were performed using FujiFilm, ImageJ software; Version: 2.0.0-rc-41/1.5d (Fuji, Minato, Tokyo, Japan). Change in fluorescence was calculated by the following equation: Δ*F*=(*F*−*F*_0_)/(*F*_max_−*F*_0_), where *F* is the fluorescence intensity at any given time, *F*_0_ is the baseline fluorescence intensity, and *F*_max_ is the maximum fluorescence intensity when exposed to ionomycin (10 *μ*M).

### Mitochondrial permeability transition pore

Astrocytes were cultured in 96-well plates at 0.05×10^6^ cells/well. After 24 h treatment, the calcein/cobalt chloride quenching technique was used to elucidate mPTP opening using image-iT LIVE mitochondrial transition pore assay kit (Thermo Fisher Scientific). Live cells were imaged with appropriate excitation and emission filters for fluorescein on ECLIPSE Ti-4 using the NIS-Elements BR. 3.2 software (Nikon). In closed mPTP condition, cobalt quenches calcein fluorescence not sequestered in mitochondria; therefore, colocalization of MTR reflected as green/yellow for mitochondria.

## Additional Information

**Publisher’s note:** Springer Nature remains neutral with regard to jurisdictional claims in published maps and institutional affiliations.

## References

[bib1] Elbirt D, Mahlab-Guri K, Bezalel-Rosenberg S, Gill H, Attali M, Asher I. HIV-associated neurocognitive disorders (HAND). Isr Med Assoc J 2015; 17: 54–59. 25739180

[bib2] Matinella A, Lanzafame M, Bonometti MA, Gajofatto A, Concia E, Vento S et al. Neurological complications of HIV infection in pre-HAART and HAART era: a retrospective study. J Neurol 2015; 262: 1317–1327. 25877836 10.1007/s00415-015-7713-8

[bib3] Achim CL, Heyes MP, Wiley CA. Quantitation of human immunodeficiency virus, immune activation factors, and quinolinic acid in AIDS brains. J Clin Invest 1993; 91: 2769–2775. 8514884 10.1172/JCI116518 PMC443343

[bib4] Bagetta G, Corasaniti MT, Berliocchi L, Nistico R, Giammarioli AM, Malorni W et al. Involvement of interleukin-1beta in the mechanism of human immunodeficiency virus type 1 (HIV-1) recombinant protein gp120-induced apoptosis in the neocortex of rat. Neuroscience 1999; 89: 1051–1066. 10362294 10.1016/s0306-4522(98)00363-7

[bib5] Gendelman HE, Lipton SA, Tardieu M, Bukrinsky MI, Nottet HS. The neuropathogenesis of HIV-1 infection. J Leukoc Biol 1994; 56: 389–398. 8083614 10.1002/jlb.56.3.389

[bib6] Underwood J, Robertson KR, Winston A. Could antiretroviral neurotoxicity play a role in the pathogenesis of cognitive impairment in treated HIV disease? AIDS 2015; 29: 253–261. 25426811 10.1097/QAD.0000000000000538

[bib7] Robertson KR, Su Z, Margolis DM, Krambrink A, Havlir DV, Evans S et al. Neurocognitive effects of treatment interruption in stable HIV-positive patients in an observational cohort. Neurology 2010; 74: 1260–1266. 20237308 10.1212/WNL.0b013e3181d9ed09 PMC2860482

[bib8] Brown MK, Naidoo N. The endoplasmic reticulum stress response in aging and age-related diseases. Front Physiol 2012; 3: 263. 22934019 10.3389/fphys.2012.00263 PMC3429039

[bib9] Bellucci A, Navarria L, Zaltieri M, Falarti E, Bodei S, Sigala S et al. Induction of the unfolded protein response by alpha-synuclein in experimental models of Parkinson's disease. J Neurochem 2011; 116: 588–605. 21166675 10.1111/j.1471-4159.2010.07143.x

[bib10] Kaufman RJ. Orchestrating the unfolded protein response in health and disease. J Clin Invest 2002; 110: 1389–1398. 12438434 10.1172/JCI16886 PMC151822

[bib11] Schroder M. Endoplasmic reticulum stress responses. Cell Mol Life Sci 2008; 65: 862–894. 18038217 10.1007/s00018-007-7383-5 PMC11131897

[bib12] Andras IE, Toborek M. Amyloid beta accumulation in HIV-1-infected brain: the role of the blood brain barrier. IUBMB Life 2013; 65: 43–49. 23225609 10.1002/iub.1106 PMC3804005

[bib13] Achim CL, Adame A, Dumaop W, Everall IP, Masliah E, Neurobehavioral Research C. Increased accumulation of intraneuronal amyloid beta in HIV-infected patients. J Neuroimmune Pharmacol 2009; 4: 190–199. 19288297 10.1007/s11481-009-9152-8 PMC3055557

[bib14] Lindl KA, Akay C, Wang Y, White MG, Jordan-Sciutto KL. Expression of the endoplasmic reticulum stress response marker, BiP, in the central nervous system of HIV-positive individuals. Neuropathol Appl Neurobiol 2007; 33: 658–669. 17931354 10.1111/j.1365-2990.2007.00866.x

[bib15] Ton H, Xiong H. Astrocyte Dysfunctions and HIV-1 Neurotoxicity. J AIDS Clin Res 2013; 4: 255. 24587966 10.4172/2155-6113.1000255 PMC3938291

[bib16] Sokka AL, Putkonen N, Mudo G, Pryazhnikov E, Reijonen S, Khiroug L et al. Endoplasmic reticulum stress inhibition protects against excitotoxic neuronal injury in the rat brain. J Neurosci 2007; 27: 901–908. 17251432 10.1523/JNEUROSCI.4289-06.2007 PMC6672923

[bib17] Dandekar A, Mendez R, Zhang K. Cross talk between ER stress, oxidative stress, and inflammation in health and disease. Methods Mol Biol 2015; 1292: 205–214. 25804758 10.1007/978-1-4939-2522-3_15

[bib18] Shah A, Vaidya NK, Bhat HK, Kumar A. HIV-1 gp120 induces type-1 programmed cell death through ER stress employing IRE1alpha, JNK and AP-1 pathway. Sci Rep 2016; 6: 18929. 26740125 10.1038/srep18929 PMC4703964

[bib19] Fan Y, He JJ. HIV-1 Tat induces unfolded protein response and endoplasmic reticulum stress in astrocytes and causes neurotoxicity through glial fibrillary acidic protein (GFAP) activation and aggregation. J Biol Chem 2016; 291: 22819–22829. 27609520 10.1074/jbc.M116.731828 PMC5077214

[bib20] Kang DC, Su ZZ, Sarkar D, Emdad L, Volsky DJ, Fisher PB. Cloning and characterization of HIV-1-inducible astrocyte elevated gene-1, AEG-1. Gene 2005; 353: 8–15. 15927426 10.1016/j.gene.2005.04.006

[bib21] Vartak-Sharma N, Ghorpade A. Astrocyte elevated gene-1 regulates astrocyte responses to neural injury: implications for reactive astrogliosis and neurodegeneration. J Neuroinflammation 2012; 9: 195. 22884085 10.1186/1742-2094-9-195 PMC3488579

[bib22] Carnemolla A, Fossale E, Agostoni E, Michelazzi S, Calligaris R, De Maso L et al. Rrs1 is involved in endoplasmic reticulum stress response in Huntington disease. J Biol Chem 2009; 284: 18167–18173. 19433866 10.1074/jbc.M109.018325 PMC2709382

[bib23] Brabers NA, Nottet HS. Role of the pro-inflammatory cytokines TNF-alpha and IL-1beta in HIV-associated dementia. Eur J Clin Invest 2006; 36: 447–458. 16796601 10.1111/j.1365-2362.2006.01657.x

[bib24] Moynagh PN. The interleukin-1 signalling pathway in astrocytes: a key contributor to inflammation in the brain. J Anat 2005; 207: 265–269. 16185251 10.1111/j.1469-7580.2005.00445.x PMC1571539

[bib25] Sundaram M, Saghayam S, Priya B, Venkatesh KK, Balakrishnan P, Shankar EM et al. Changes in antioxidant profile among HIV-infected individuals on generic highly active antiretroviral therapy in southern India. Int J Infect Dis 2008; 12: e61–e66. 18621564 10.1016/j.ijid.2008.04.004

[bib26] Apostolova N, Funes HA, Blas-Garcia A, Alegre F, Polo M, Esplugues JV. Involvement of nitric oxide in the mitochondrial action of efavirenz: a differential effect on neurons and glial cells. J Infect Dis 2015; 211: 1953–1958. 25538272 10.1093/infdis/jiu825

[bib27] Nwogu JN, Ma Q, Babalola CP, Adedeji WA, Morse GD, Taiwo B. Pharmacokinetic, pharmacogenetic, and other factors influencing CNS penetration of antiretrovirals. AIDS Res Treat 2016; 2016: 2587094. 27777797 10.1155/2016/2587094 PMC5061948

[bib28] Baird TD, Wek RC. Eukaryotic initiation factor 2 phosphorylation and translational control in metabolism. Adv Nutr 2012; 3: 307–321. 22585904 10.3945/an.112.002113 PMC3649462

[bib29] Chen TW, Wardill TJ, Sun Y, Pulver SR, Renninger SL, Baohan A et al. Ultrasensitive fluorescent proteins for imaging neuronal activity. Nature 2013; 499: 295–300. 23868258 10.1038/nature12354 PMC3777791

[bib30] Ellgaard L, Helenius A. Quality control in the endoplasmic reticulum. Nat Rev Mol Cell Biol 2003; 4: 181–191. 12612637 10.1038/nrm1052

[bib31] Thirkettle HJ, Girling J, Warren AY, Mills IG, Sahadevan K, Leung H et al. LYRIC/AEG-1 is targeted to different subcellular compartments by ubiquitinylation and intrinsic nuclear localization signals. Clin Cancer Res 2009; 15: 3003–3013. 19383828 10.1158/1078-0432.CCR-08-2046

[bib32] Vartak-Sharma N, Nooka S, Ghorpade A. Astrocyte elevated gene-1 (AEG-1) and the A(E)Ging HIV/AIDS-HAND. Prog Neurobiol 2017; 157: 133–157. 27090750 10.1016/j.pneurobio.2016.03.006 PMC5982115

[bib33] Gorlach A, Bertram K, Hudecova S, Krizanova O. Calcium and ROS: a mutual interplay. Redox Biol 2015; 6: 260–271. 26296072 10.1016/j.redox.2015.08.010 PMC4556774

[bib34] Wesselingh SL, Power C, Glass JD, Tyor WR, McArthur JC, Farber JM et al. Intracerebral cytokine messenger RNA expression in acquired immunodeficiency syndrome dementia. Ann Neurol 1993; 33: 576–582. 8498837 10.1002/ana.410330604

[bib35] Wesselingh SL, Glass J, McArthur JC, Griffin JW, Griffin DE. Cytokine dysregulation in HIV-associated neurological disease. Adv Neuroimmunol 1994; 4: 199–206. 7874388 10.1016/s0960-5428(06)80258-5

[bib36] Garg AD, Kaczmarek A, Krysko O, Vandenabeele P, Krysko DV, Agostinis P. ER stress-induced inflammation: does it aid or impede disease progression? Trends Mol Med 2012; 18: 589–598. 22883813 10.1016/j.molmed.2012.06.010

[bib37] Li Y, Schwabe RF, DeVries-Seimon T, Yao PM, Gerbod-Giannone MC, Tall AR et al. Free cholesterol-loaded macrophages are an abundant source of tumor necrosis factor-alpha and interleukin-6: model of NF-kappaB- and map kinase-dependent inflammation in advanced atherosclerosis. J Biol Chem 2005; 280: 21763–21772. 15826936 10.1074/jbc.M501759200

[bib38] Zhang K, Kaufman RJ. From endoplasmic-reticulum stress to the inflammatory response. Nature 2008; 454: 455–462. 18650916 10.1038/nature07203 PMC2727659

[bib39] Verfaillie T, Garg AD, Agostinis P. Targeting ER stress induced apoptosis and inflammation in cancer. Cancer Lett 2013; 332: 249–264. 20732741 10.1016/j.canlet.2010.07.016

[bib40] Decloedt EH, Rosenkranz B, Maartens G, Joska J. Central nervous system penetration of antiretroviral drugs: pharmacokinetic, pharmacodynamic and pharmacogenomic considerations. Clin Pharmacokinet 2015; 54: 581–598. 25777740 10.1007/s40262-015-0257-3

[bib41] Dybul M, Fauci AS, Bartlett JG, Kaplan JE, Pau AK, Panel on Clinical Practices for the Treatment of HIV. Guidelines for using antiretroviral agents among HIV-infected adults and adolescents. Recommendations of the Panel on Clinical Practices for Treatment of HIV. MMWR Recomm Rep 2002; 51 (RR-7): 1–55. 12027060

[bib42] Robertson K, Liner J, Meeker RB. Antiretroviral neurotoxicity. J Neurovirol 2012; 18: 388–399. 22811264 10.1007/s13365-012-0120-3 PMC3581315

[bib43] Li Y, Guo Y, Tang J, Jiang J, Chen Z. New insights into the roles of CHOP-induced apoptosis in ER stress. Acta Biochim Biophys Sin (Shanghai) 2014; 46: 629–640. 25016584 10.1093/abbs/gmu048

[bib44] Rutkowski DT, Arnold SM, Miller CN, Wu J, Li J, Gunnison KM et al. Adaptation to ER stress is mediated by differential stabilities of pro-survival and pro-apoptotic mRNAs and proteins. PLoS Biol 2006; 4: e374. 17090218 10.1371/journal.pbio.0040374 PMC1634883

[bib45] Akay C, Lindl KA, Shyam N, Nabet B, Goenaga-Vazquez Y, Ruzbarsky J et al. Activation status of integrated stress response pathways in neurones and astrocytes of HIV-associated neurocognitive disorders (HAND) cortex. Neuropathol Appl Neurobiol 2012; 38: 175–200. 21883374 10.1111/j.1365-2990.2011.01215.x PMC3708539

[bib46] Su ZZ, Kang DC, Chen Y, Pekarskaya O, Chao W, Volsky DJ et al. Identification and cloning of human astrocyte genes displaying elevated expression after infection with HIV-1 or exposure to HIV-1 envelope glycoprotein by rapid subtraction hybridization, RaSH. Oncogene 2002; 21: 3592–3602. 12032861 10.1038/sj.onc.1205445

[bib47] Hu XT. HIV-1 Tat-mediated calcium dysregulation and neuronal dysfunction in vulnerable brain regions. Curr Drug Targets 2016; 17: 4–14. 26028040 10.2174/1389450116666150531162212 PMC4772427

[bib48] Haughey NJ, Mattson MP. Calcium dysregulation and neuronal apoptosis by the HIV-1 proteins Tat and gp120. J Acquir Immune Defic Syndr 2002; 31(Suppl 2): S55–S61. 12394783 10.1097/00126334-200210012-00005

[bib49] Verkhratsky A. Physiology and pathophysiology of the calcium store in the endoplasmic reticulum of neurons. Physiol Rev 2005; 85: 201–279. 15618481 10.1152/physrev.00004.2004

[bib50] Lievremont JP, Rizzuto R, Hendershot L, Meldolesi J. BiP, a major chaperone protein of the endoplasmic reticulum lumen, plays a direct and important role in the storage of the rapidly exchanging pool of Ca2+. J Biol Chem 1997; 272: 30873–30879. 9388233 10.1074/jbc.272.49.30873

[bib51] Tsujimoto Y, Shimizu S. Role of the mitochondrial membrane permeability transition in cell death. Apoptosis 2007; 12: 835–840. 17136322 10.1007/s10495-006-0525-7

[bib52] Rasola A, Bernardi P. Mitochondrial permeability transition in Ca(2+)-dependent apoptosis and necrosis. Cell Calcium 2011; 50: 222–233. 21601280 10.1016/j.ceca.2011.04.007

[bib53] Malarkey EB, Parpura V. Mechanisms of glutamate release from astrocytes. Neurochem Int 2008; 52: 142–154. 17669556 10.1016/j.neuint.2007.06.005 PMC2267911

[bib54] Bezzi P, Carmignoto G, Pasti L, Vesce S, Rossi D, Rizzini BL et al. Prostaglandins stimulate calcium-dependent glutamate release in astrocytes. Nature 1998; 391: 281–285. 9440691 10.1038/34651

[bib55] Gardner J, Borgmann K, Deshpande MS, Dhar A, Wu L, Persidsky R et al. Potential mechanisms for astrocyte-TIMP-1 downregulation in chronic inflammatory diseases. J Neurosci Res 2006; 83: 1281–1292. 16555295 10.1002/jnr.20823

[bib56] Heinzinger N, Baca-Regen L, Stevenson M, Gendelman HE. Efficient synthesis of viral nucleic acids following monocyte infection by HIV-1. Virology 1995; 206: 731–735. 7831833 10.1016/s0042-6822(95)80097-2 PMC9513716

[bib57] Chadderton T, Wilson C, Bewick M, Gluck S. Evaluation of three rapid RNA extraction reagents: relevance for use in RT-PCR's and measurement of low level gene expression in clinical samples. Cell Mol Biol (Noisy-le-grand) 1997; 43: 1227–1234. 9489949

